# Cross-sectional imaging of common and unusual complications after endoscopic retrograde cholangiopancreatography

**DOI:** 10.1007/s13244-015-0393-1

**Published:** 2015-02-26

**Authors:** Massimo Tonolini, Alessandra Pagani, Roberto Bianco

**Affiliations:** Department of Radiology, “Luigi Sacco” University Hospital, Via G.B. Grassi 74, 20157 Milan, Italy

**Keywords:** Endoscopic retrograde cholangiopancreatography (ERCP), Complications, Computed tomography (CT), Acute pancreatitis, Duodenal perforation

## Abstract

Endoscopic retrograde cholangiopancreatography (ERCP) is currently a primarily therapeutic procedure that is extensively employed to treat several biliopancreatic disorders. Although widely considered a safe procedure, ERCP is associated with a non-negligible morbidity and occasional mortality. Due to the number and complexity of operative ERCPs performed, radiologists are increasingly faced with urgent requests for investigation of suspected post-procedural complications, which often have similar clinical and laboratory manifestations. This pictorial essay reviews the usual post-procedural CT findings, the clinical features and imaging appearances of common and unusual post-ERCP occurrences including interstitial oedematous and necrotising acute pancreatitis, haemorrhages, retroperitoneal and intraperitoneal duodenal perforations, infections and stent-related complications. Emphasis is placed on the pivotal role of multidetector CT, which is warranted after complex or prolonged ERCP procedures as it represents the most effective modality to detect and grade ERCP-related complications and to monitor nonsurgically treated patients. Timely diagnosis and optimal management require a combination of clinical and laboratory data with imaging appearances; therefore, this article aims to provide an increased familiarity with interpretation of early post-ERCP studies, particularly to triage those occurrences that require interventional or surgical treatment. In selected patients MRI allows imaging pancreatitis and abnormal collections without the use of ionising radiation.

*Teaching Points*

• *Endoscopic retrograde cholangiopancreatography (ERCP) allows treating many biliopancreatic disorders*.

• *Due to the number and complexity of procedures, post-ERCP complications are increasingly encountered*.

• *Main complications include acute pancreatitis, haemorrhages, duodenal perforation and infections*.

• *Diagnosis and management of complications rely on combined clinical, laboratory and imaging data*.

• *Multidetector CT is most effective to diagnose, categorise and monitor post-ERCP complications*.

## Introduction

### Background

Due to the widespread availability of non-invasive imaging techniques such as magnetic resonance cholangiopancreatography (MRCP) and multidetector computed tomography (CT), during the last decade endoscopic retrograde cholangiopancreatography (ERCP) has evolved from a diagnostic tool towards a primarily therapeutic procedure. Currently indispensable in surgical practice, ERCP is extensively used to treat several disorders of the biliopancreatic system so that over 500,000 procedures are performed yearly in the USA [1].

The growing number of indications for ERCP encompass retained or recurrent choledocholithiasis, benign and malignant biliary strictures, ampullary stenosis, sphincter of Oddi dysfunction (SOD), postoperative and traumatic ductal injuries, acute gallstone and chronic pancreatitis. Widely considered a safe procedure that often obviates the need for surgery (particularly in frail patients with poor performance status), ERCP has limited contraindications such as upper aerodigestive obstruction, severe coagulopathy, oesophageal and/or gastric varices, anaphylactic reaction to iodinated contrast medium (CM), acute nonbiliary pancreatitis, severe cardiopulmonary impairment and recent myocardial infarction. ERCP is technically challenging in patients with pancreatico-duodenectomy or Billroth type II surgical reconstruction, unfeasible with the previous Roux-en-Y anastomosis. Following identification and cannulation of the ampullary orifice and CM injection under fluoroscopy, current endoscopic equipment allows performing sphincterotomy, extraction of common bile duct (CBD) stones, lithotripsy, biliary drainage, stricture dilatation, brush cytology and biopsy [1–3].

Furthermore, endoscopic positioning of biliary prostheses allows minimally invasive management of obstructive jaundice from benign and neoplastic causes with high technical (over 90 %) and clinical (approximately 80 %) success rates. Plastic stents are commonly used to treat benign strictures, postoperative bile leaks and pancreatic diseases. Conversely, self-expanding metal stents (SEMS) may provide effective palliation of malignant biliary obstruction [4, 5].

However, ERCP is associated with a non-negligible post-procedural morbidity (estimated in the range 4–10 % overall). The most common short-term (occurring within 3 days) complications include more or less severe cardiopulmonary problems (hypoxia, arrhythmia and aspiration) commonly associated with medications used for sedation and analgesia. The reported incidence of ERCP-specific complications ranges from 5 to 40 %, depending on the underlying diagnosis, patient age and comorbidities, complexity of the procedure, and operator experience. The most common occurrences include post-ERCP acute pancreatitis (PEAP, 2–9 %), haemorrhage (1.3–3.7 %), infection (1.9–3.6 %) and duodenal perforation (DP) in descending order of frequency. Additionally, a variety of rare complications have been occasionally reported, including pneumothorax, portal venous air embolism, splenic injury and perforation of colonic diverticula [2, 6–9].

The risk of complications is increased by operative techniques such as use of balloons and dilating catheters, tissue sampling, mechanical lithotripsy and wire baskets for stone extraction, and plastic and metallic biliary stents. Mostly related to operative procedures, ERCP-related mortality (0.5–1.4 %) may result from any of the above-mentioned complications and is particularly high in elderly patients with comorbidities and in centres with limited caseloads [2, 6–8, 10].

### Aim

According to the guidelines issued by the American Society for Gastrointestinal Endoscopy (ASGE) and the World Society of Emergency Surgery (WSES), diagnosis and management of ERCP-related complications should rely upon a combination of clinical, laboratory and imaging data. Since iatrogenic complications represent a non-negligible cause of litigation and lawsuits, early recognition and prompt appropriate intervention are essential in optimising patient management and outcome [6, 11, 12].

Based upon 8 years of personal experience at a university-based general hospital where approximately 230 ERCPs are performed each year, this pictorial essay reviews and illustrates the normal post-procedural appearances observed shortly after ERCP, and the imaging findings associated with common and unusual complications, aiming to provide radiologists with an increased familiarity with interpretation of early post-procedural studies for an optimal triage of complications. Most of the emphasis is placed on the pivotal role of multidetector CT, which represents, according to the WSES guidelines for management of intra-abdominal infections, the preferred and most effective modality to comprehensively investigate the abdomen and pelvis to search for possible iatrogenic complications [6, 12–15].

## Early post-ERCP imaging: indications, techniques, usual findings and reactive changes

### Clinical features and indications for imaging

In the vast majority of cases, the clinical suspicion of early iatrogenic complications is based on a combination of intraprocedural findings (difficult or repeated cannulation, suspected or confirmed DP), symptoms and physical signs (sudden or worsening abdominal pain, distension, fever, haemodynamic impairment) and laboratory data (decreasing haemoglobin, elevated leukocyte count, acute phase reactants, serum lipase or amylase). The key role of CT imaging to investigate patients who become acutely ill hours or days after ERCP results from the limited value of physical examination (due to the retroperitoneal location of most iatrogenic changes) and the similar clinical and laboratory manifestations observed in most complications, particularly AP and DP [2, 6, 12].

Furthermore, ERCP duration may represent a surrogated marker for challenging cannulation and/or operative manoeuvres that can lead to papillary oedema and compromise ductal outflow, thus resulting in an increased risk and severity of complications, particularly PEAP. According to both the literature and personal experience, planned CT may be warranted in patients after prolonged ERCP procedures, particularly with sphincterotomy or multiple cannulations [16].

### CT technique, usual post-procedural findings and reactive changes

**Fig. 1:**
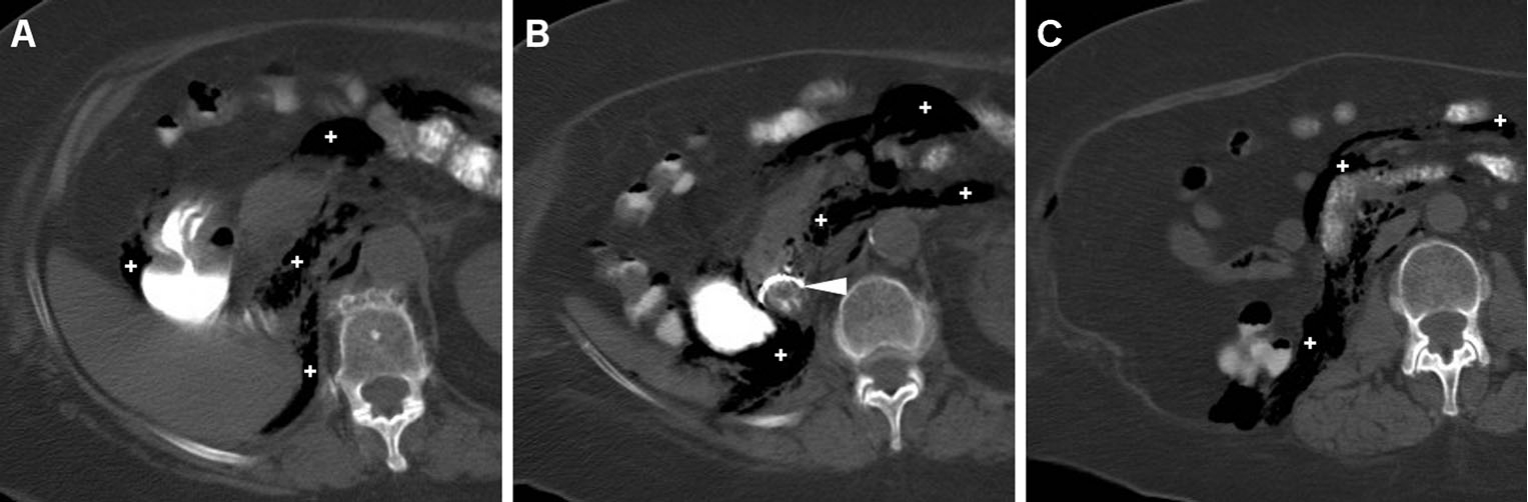
During endoscopic retrograde cholangiopancreatography (*ERCP*), a 65-year-old female with sphincter of Oddi dysfunction experienced duodenal perforation (*DP*), which was recognised intraprocedurally and treated with endoluminal clipping. Twenty-four hours later, unenhanced multidetector CT obtained after peroral ingestion of enteral contrast medium (*CM*) and viewed at the bone window setting showed a well-opacified gastroduodenal lumen without extraluminal CM leak, consistent with sealed DP. Note the persistent extensive retroperitoneal air (+) and metallic clips (*arrowhead in B*)

A preliminary unenhanced CT acquisition without intravenous and peroral CM is helpful to visualise extraluminal air (particularly using image review at lung or bone window settings) and hyperattenuating (35–70 Hounsfield units, HU) fresh blood. Afterwards, following standard-dose intravenous CM injection we recommend a biphasic (arterial-dominant and venous) acquisition, particularly to identify pancreatic necrosis and CM extravasation indicating active bleeding. Additionally, in selected cases gastroduodenal opacification by means of peroral ingestion of diluted enteral CM may be beneficial to confirm or exclude leakage indicating DP (Fig. 1) [6, 13–15].

**Fig. 2:**
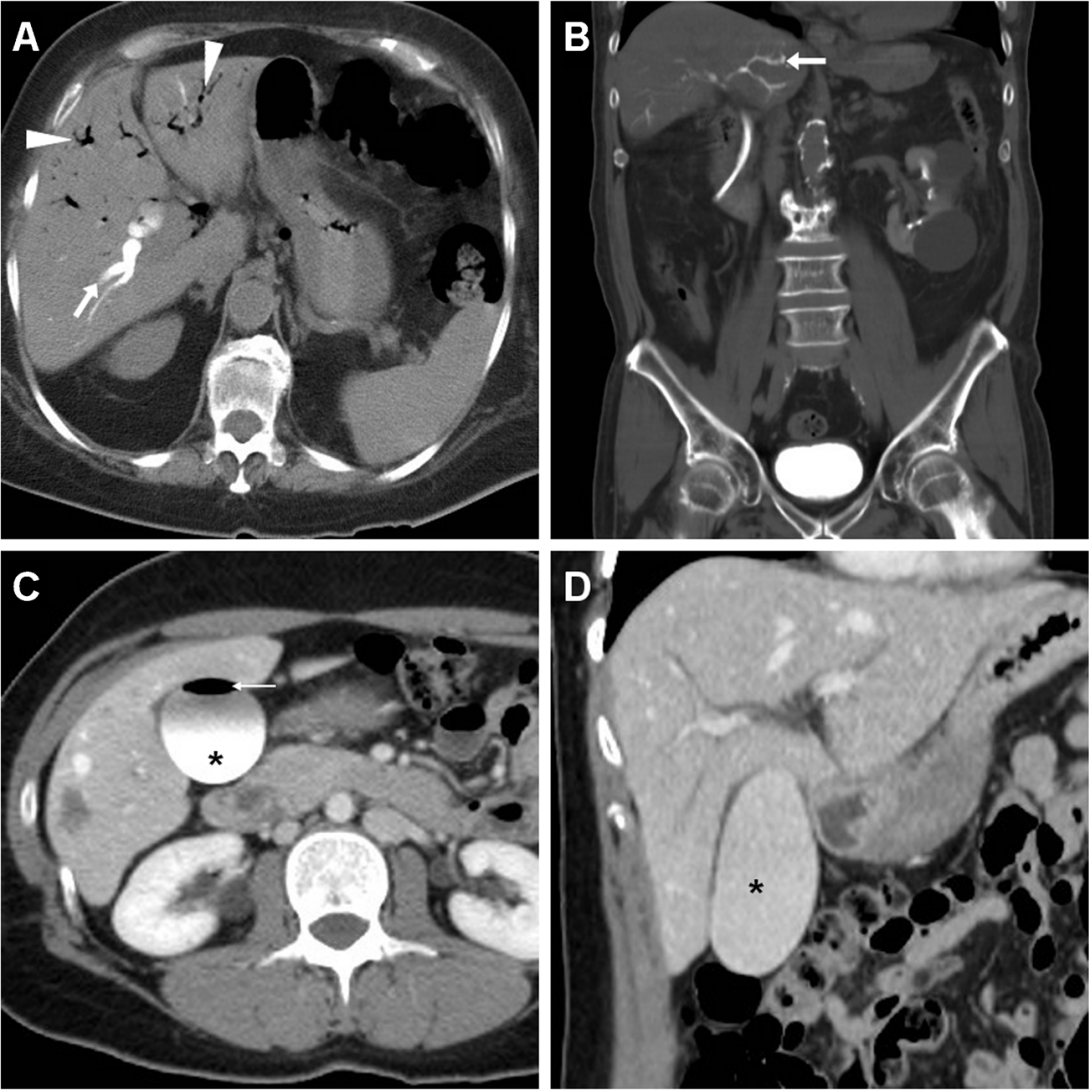
Normal findings observed hours after operative ERCP in a 75-year-old male investigated with unenhanced CT (**c**, **d**) to verify correct placement of a plastic biliary stent, including dependent contrast medium (*arrows*) and air (*arrowheads*) in the intrahepatic bile ducts. In a different patient, a 44-year-old female patient suffering from acute post-procedural pain, contrast-enhanced CT (**a**, **b**) 2 days after ERCP excluded signs of acute complications and showed stratified iodinated contrast medium (*), bile and air (*thin arrow*) in the gallbladder

After ERCP the presence of air in the biliary tree is an expected, common finding. Intra- and extrahepatic pneumobilia is visible in the majority of patients studied with CT within a few weeks from the procedure and may persist for months or years in patients who underwent sphincterotomy (Fig. 2). Shortly after ERCP retained CM is commonly seen in the biliary tree and gallbladder, often with a characteristic stratified appearance (Fig. 2) [13, 15].

**Fig. 3:**
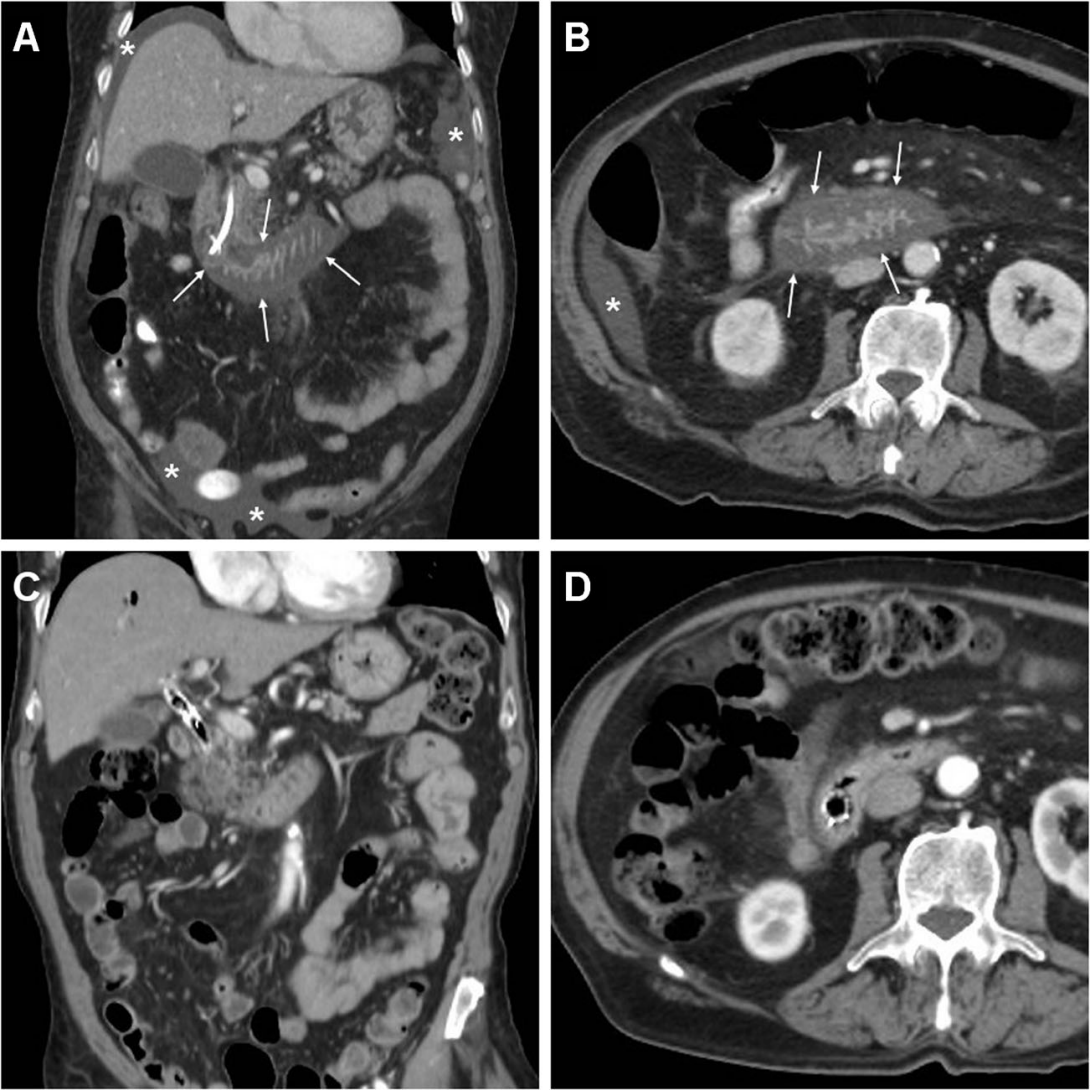
Acute reversible duodenitis following ERCP. A 78-year-old male with common bile duct (*CBD*) carcinoma causing concentric stenosis of the proximal choledochus suffered from acute diffuse abdominal pain without peritonitis 24 h after ERCP including cytological brushing and positioning of a 10-French plastic biliary stent. Urgent contrast-enhanced CT (**a**, **b**) showed a correctly placed biliary stent, appearance of moderate ascites (*) and of marked circumferential thickening of the duodenum from the Vaterian papilla to the Treitz angle, with enhancing mucosa and hypoattenuating oedematous submucosa (“*target sign*”, *thin arrows*), and minimal associated inflammatory changes in the periduodenal fat. Imaging findings, laboratory data and the subsequent course excluded iatrogenic pancreatitis, haemorrhage and DP. Three weeks later, the plastic biliary stent was removed and replaced with a self-expanding metal stent (*SEMS*). Follow-up CT (**d**) showed imaging resolution of both biliary obstruction and acute duodenal inflammatory changes [partly reprinted from Ref. 20]

Multiplanar CT reformations (Figs. 2 and 3) provide high-resolution visualisation of biliary endoprostheses, whether of plastic or metallic reticular “mesh” material, and of their anatomical relationships. When interpreting post-procedural studies, the stent type should be reported along with the integrity, patency and position including proximal and distal extremities [13, 17].

In up to 29 % of asymptomatic patients, limited retroperitoneal air observed within 24 h from ERCP may result from excessive insufflation during and after endoscope withdrawal. Although this occurrence is categorised as type IV perforation according to the Stapfer classification (Table 1), we suggest not to emphasise the term “perforation” in the radiological report, since its does not require treatment [8, 18, 19].

**Table 1:** Classification of duodenal perforations according to Stapfer et al. (Ref. 8)

Type	Description	Comment
I	Endoscope-related lateral or medial duodenal wall perforations	Often large and distant from the ampulla, requires surgical intervention in most cases
II	Retroperitoneal peri-Vaterian perforations resulting from (pre-cut) sphincterotomy	Of variable severity but most often discrete and amenable to conservative management
III	Distal common bile duct injuries secondary to guidewire insertion or instrumentation for stone extraction	Amenable to conservative management
IV	Isolated retroperitoneal air from excessive insufflation	Does not require specific treatment

Finally, reversible oedematous-type mural thickening consistent with acute duodenitis (Fig. 3) may be observed following therapeutic ERCP [20].

### Role and limitations of ultrasound and magnetic resonance imaging (MRI)

Compared to CT, sonographic investigation of patients with suspected post-ERCP complications is strongly limited by the usual abdominal distension due to ileus, pneumoperitoneum or pneumoretroperitoneum and by its inability to assess pancreatic necrosis and bleeding. Practically, ultrasound may be useful for rapid assessment of the gallbladder and biliary tree (with or without stents) and for follow-up of known collections, and its use is not recommended by current practice guidelines [6, 12, 14, 21].

An increasingly attractive alternative modality, MRI can image PEAP and abnormal collections without use of ionising radiation, and it is particularly beneficial in young patients. Unenhanced T1- and T2-weighted sequences with and without fat suppression may be complemented with MRCP sequences to assess the biliary and pancreatic ducts and with additional dynamic fat-suppressed T1-weighted gradient echo sequences during intravenous gadolinium contrast when pancreatic necrosis is suspected. Furthermore, diffusion-weighted MRI may be more sensitive in detecting cases of mild pancreatic inflammation. However, despite recent technical advancements MRI remains limited in acutely ill or uncooperative patients, and the presence or pneumobilia often renders MRCP sequences uninformative or confusing [13, 22, 23].

## Acute pancreatitis

Arguably the most common serious adverse event, PEAP has been reported with variable incidence (2–9 % overall, approximately 3.5 % in a meta-analysis of several prospective studies, and up to 30 % of high risk cases) according to patient selection. A transient asymptomatic increase in serum pancreatic enzymes occurs in the majority (70–75 %) of patients within 4 h after ERCP and resolves within 4 days. According to the widely used consensus definition adopted by the ASGE guidelines, diagnosis of PEAP requires at least two of the three following criteria: (1) consistent new-onset or worsening abdominal pain (persistent, severe, epigastric pain often radiating to the back); (2) new or prolongation of hospitalisation for at least 2 days; (3) abnormally elevated (at least three times above the upper normal limit) serum lipase or amylase 24 h after the procedure [2, 6, 21].

The multifactorial pathogenesis of PEAP involves mechanical factors (such as direct trauma from endoscopy, difficult bile duct cannulation and multiple pancreatic duct injections) along with enzymatic, microbiological and patient-related factors including history of allergy [24–26]. The role of iodinated CM as one of the cofactors in causing PEAP has been extensively studied. In experimental models of acute pancreatitis, digestive enzyme activation occurs rapidly within acinar cells following an initiating event: secretion blockage leads to accumulation of zymogen granules within cells, their fusion into large vacuoles, enzyme activation and finally cellular injury [24]. Besides the reduced pancreatic blood flow due to CM hyperviscosity, controversy exists on the role of the chemical effect of CM osmolality. Earlier studies reported reduced rates of pancreatic damage and decreased with newer low- or iso-osmolar compared to high-osmolar contrast medium, but other reports including a meta-analysis did not confirm a significantly different risk and outcome of PEAP [25–28].

Probably unavoidable even in the hands of experienced endoscopists, PEAP is associated with established risk conditions including patient-specific (age below 55 years, female gender, SOD, history of AP) and procedure-related factors (difficult cannulation, standard or pre-cut sphincterotomy). Whereas the risk of bleeding and perforation is similar to that of younger patients, the elderly are less likely to experience PEAP. Clinical guidelines increasingly suggest pharmacologic prophylaxis with nonsteroidal anti-inflammatory drugs, glyceryl trinitrate or somatostatin; furthermore, in patients with SOD, the use of temporary pancreatic duct stenting has been proposed to limit the risk and severity of PEAP. Treatment is analogous to that of non-iatrogenic AP, and mortality reaches 3 % of cases [2, 6, 21].

In patients with consistent above-mentioned clinical and laboratory criteria following ERCP, early imaging is generally unnecessary to diagnose PEAP, and during the first week organ failure and systemic inflammatory response syndrome (SIRS) may be present with few local complications. Although not specifically developed for iatrogenic occurrences, the revised Atlanta classification differentiates morphologically interstitial oedematous (IEP) from necrotising acute pancreatitis (NAP), the latter associated with a high probability of infection, subsequent organ failure and increased mortality (12–30 % versus below 3 %). CT diagnosis of IEP relies on diffuse or localised enlargement of the pancreatic parenchyma with preserved homogeneous enhancement (to 80–150 HU) after intravenous CM, accompanied by peripancreatic fat stranding or by hypoattenuating (0–30 HU) acute peripancreatic fluid collections (APFCs). Containing a mixture of exudates, necrosis and blood, APFCs are adjacent to the pancreas and confined by normal fascial planes, and they display homogeneous low (0–30 HU) attenuation without a discernible wall and enhancement [22, 23].

**Fig. 4:**
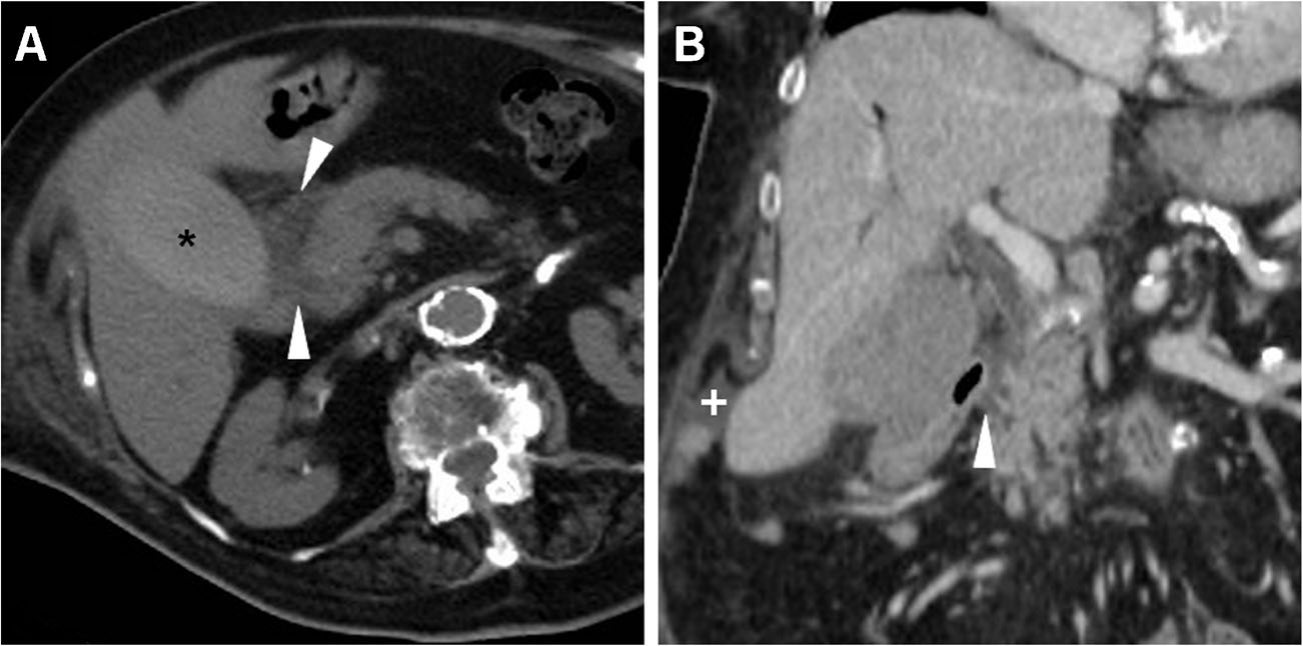
Oedematous acute pancreatitis (*AP*) in a 77-year-old female 3 days after endoscopic treatment of choledocholithiasis including sphincterotomy. Unenhanced (**a**) and post-contrast (**b**) CT images showed persistent moderately hyperattenuating contrast medium (*) in the gallbladder, oedema and fluid in the pancreatico-duodenal groove (*arrows*), minimal ascites (+) consistent with clinical and laboratory features of mild AP, and excluded abnormal collections and non-enhancing parenchyma indicating pancreatic necrosis. The patient had an uneventful course with conservative treatment

**Fig. 5:**
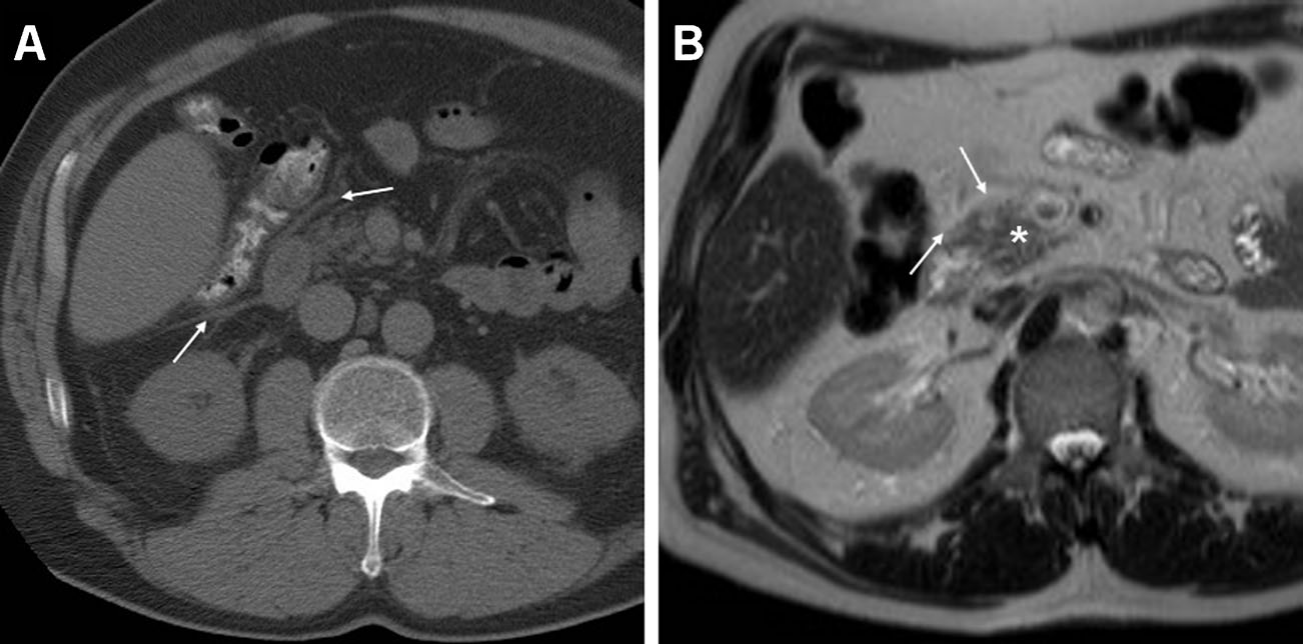
Oedematous AP with markedly increased serum lipase 48 h after ERCP treatment of choledocholithiasis in a 55-year-old male with previous history of biliary AP and laparoscopic cholecystectomy. Unenhanced CT (**a**) and T2-weighted MRI (**b**) images show mild peripancreatic and retroperitoneal fascial fluid (*thin arrows*) without abnormal collections. Clinical symptoms and laboratory changes regressed within 72 h

Nevertheless, in our experience CT is requested within 24–72 h in most patients with clinical and laboratory features indicating PEAP to exclude other complications (particularly DP) with similar manifestations. Therefore, a majority of patients with IEP are imaged with near-normal or subtle CT findings, including a homogeneously enhancing pancreas, fat stranding and minimal fluid around the head and neck of the pancreas (Figs. 4 and 5). Borrowing from experience with gallstone AP, early contrast-enhanced CT obtained within 2–3 days from the onset of symptoms may underestimate the severity of PEAP including confident exclusion of necrosis so that repeated scanning may be required during post-ERCP hospitalisation [13, 15, 16, 22, 23].

**Fig. 6:**
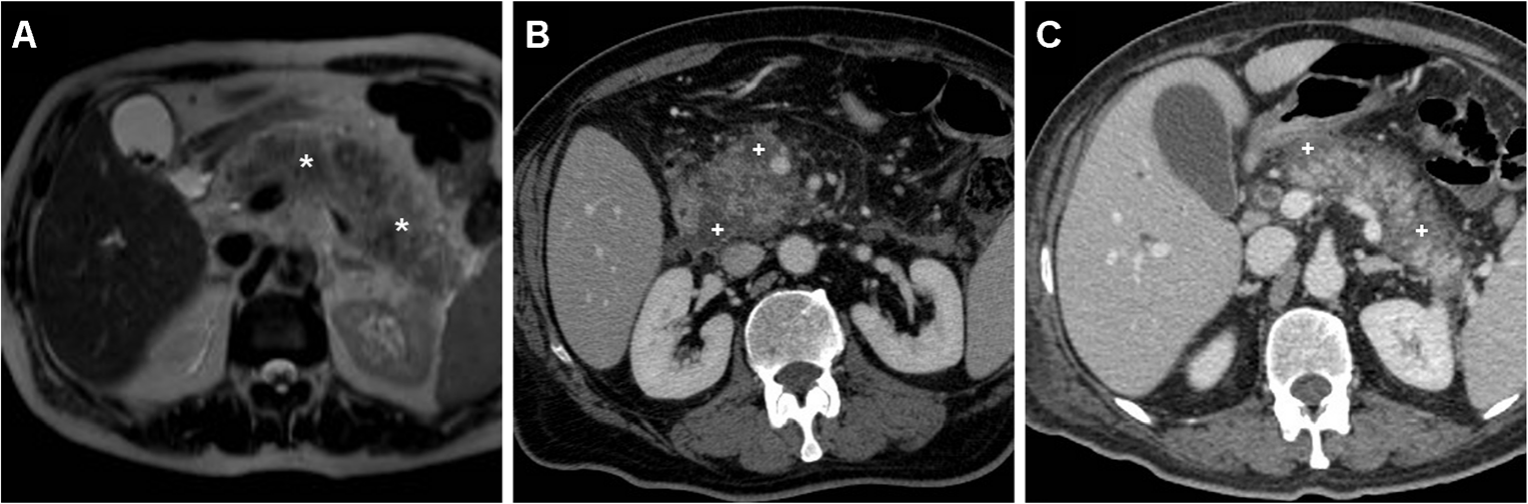
Necrotising AP in a 73-year-old male after ERCP including endoscopic treatment of mild bleeding after papillotomy. Urgent MRI (T2-weighted image in **a**) requested because of increased C-reactive protein (*CRP*), leukocyte count and serum lipase showed a markedly enlarged pancreatic gland (*) with peripancreatic oedema. Contrast-enhanced CT (**b**, **c**) confirmed severe AP with inhomogeneous enhancement of the enlarged pancreas due to diffuse peripancreatic necrosis (+). The patient finally recovered after 2 weeks of in-hospital conservative treatment

**Fig. 7:**
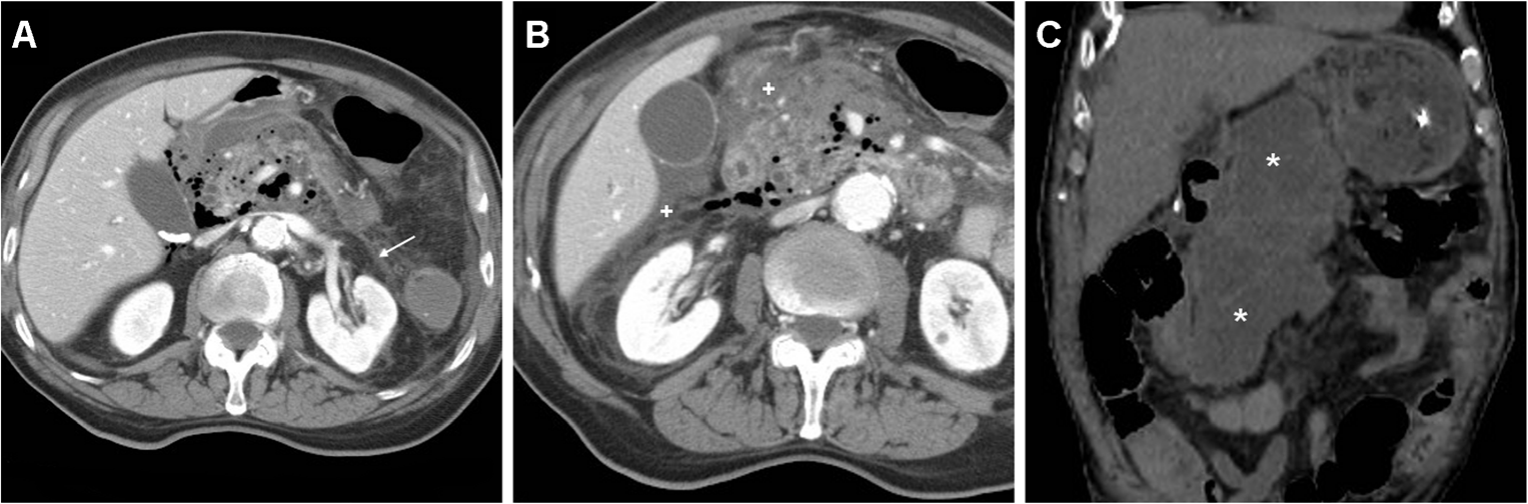
Necrotising AP plus retroperitoneal air in a 79-year-old male occurring 48 h after ERCP treatment of choledocholithiasis, including sphincterotomy. Contrast-enhanced CT (**a**, **b**) showed an enlarged, markedly inhomogeneous, poorly enhancing pancreas with fascial (*thin arrow* in **a**), peripancreatic and infrahepatic (+ in **b**) fluid, gas bubbles along the superior mesenteric vessels and in the periduodenal region. Pleural effusion was present bilaterally. Urgent laparotomic surgery confirmed severe necrotic-haemorrhagic pancreatitis. During his prolonged stay in the intensive care unit, follow-up unenhanced CT (**c**) showed the appearance of a vast walled-off postnecrotic collection (*)

Conversely, NAP includes fatty tissue necrosis in either the pancreatic parenchyma or peripancreatic tissue and corresponds at imaging to pancreatic enlargement, heterogeneous because of single or multiple areas of diminished or absent enhancement consistent with necrosis (Figs. 6 and 7). In NAP, post-necrotic peripancreatic fluid collections (PNPFCs) are commonly observed in the pancreas, peripancreatic tissue or both. PNPFCs contain fluid, necrosis and/or loculation in varying degrees. Differentiation from APFCs is very difficult in the early phase and is generally based on the presence or absence of glandular necrosis. PNPFCs may become progressively more organised and encapsulated later and are termed walled-off pancreatic necrosis after 4 weeks of symptom onset (Fig. 7) [13, 15, 16, 22, 23].

Therefore, the radiologist’s role mostly relies in the identification of the presence and extent of necrosis. Severity of AP may be graded according to the modified CT severity index (M-CTSI), which takes into account the presence of pancreatic and/or peripancreatic inflammatory changes, APFCs, necrosis (<30 %, 30–50 % and over 50 %) and extrapancreatic complications such as pleural effusion, ascites, vascular or gastrointestinal involvement. The only large specific series graded PEAP severity according to M-CTSI scores as mild (≤2 points), moderate (4–6) and severe (≥8) in 53.6, 42.8 and 3.6 % of cases respectively [13, 15, 16, 22, 23].

Furthermore, peripancreatic and glandular changes consistent with PEAP are well demonstrated by MRI (Figs. 5 and 6). Typical MRI findings of AP include variable degrees of glandular enlargement with mildly increased T2-weighted signal intensity corresponding to oedema, normal signal on fat-saturated T1-weighted acquisitions, peripancreatic inflammatory fat stranding, fluid or collections. Similarly to CT, pancreatic enhancement may appear homogeneous (in IEP), diminished or heterogeneous (in NAP) after intravenous gadolinium CM. In the setting of gallstones, it has been proposed that a limited noncontrast MRI protocol has high correlation with contrast-enhanced CT in the assessment of mild AP. Furthermore, the use of diffusion-weighted MRI including measurement of apparent diffusion coefficients (ADCs) may allow differentiation among normal, inflamed and necrotic pancreas before or without CM administration [22, 23, 29, 30].

## Haemorrhage

Primarily a complication related to sphincterotomy rather than to diagnostic ERCP, bleeding may be immediate or delayed up to 2 weeks (in up to 50 % of cases) and is clinically suggested by haematemesis, melaena or haemoglobin drop. Whereas variable degrees of bleeding occur in approximately 1–2 % of treated patients, haemorrhage is graded as mild (according to blood loss and transfusion need) in approximately 70 % of cases, with occasional mortality (0.3 %). Specific risk factors include coagulopathy, therapeutic anticoagulation, haemodialysis, acute cholangitis or papillary stenosis, pre-cut or extended sphincterotomy [2, 6].

**Fig. 8:**
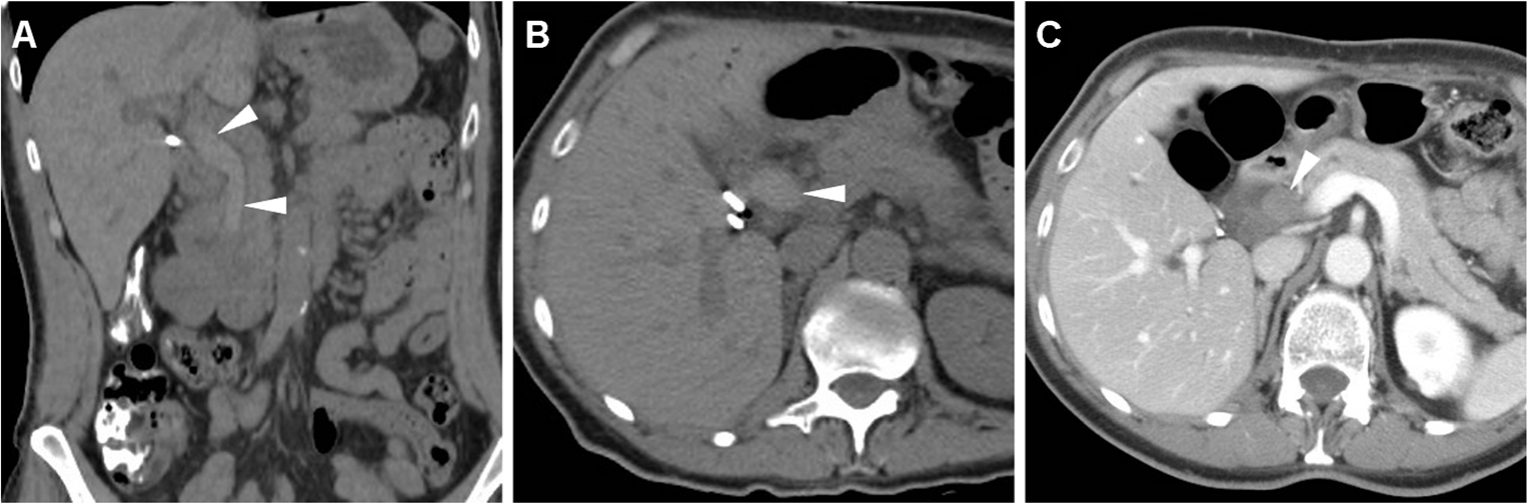
Haemobilia following ERCP in a 57-year-old female with benign papillary stenosis and previous cholecystectomy, manifesting with abdominal pain and mild haemoglobin drop. Unenhanced (**a**, **b**) and post-contrast (**c**) CT images showed moderate dilatation and hyperattenuating (45 HU) content of the CBD (*arrowheads*) corresponding to endoluminal haemorrhage but initially misinterpreted as retained CM injected during the procedure, without signs of active bleeding. This complication was self-limiting and resolved on conservative treatment

**Fig. 9:**
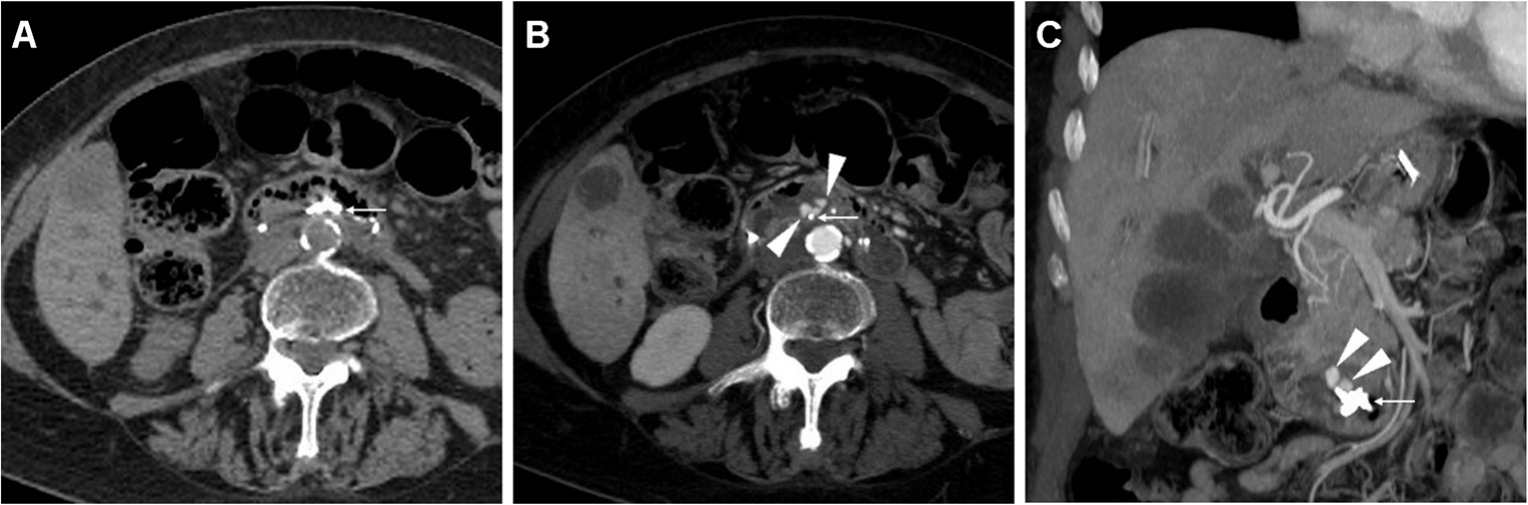
Acute intraluminal bleeding in the duodenum manifesting with acute abdominal pain and impending haemodynamic shock, hours after an unsuccessful endoscopic attempt to relieve the intrahepatic biliary obstruction in 77-year-old female with recently diagnosed infiltrating gallbladder carcinoma. Operative ERCP was interrupted after sphincterotomy because of duodenal haemorrhage, which was treated with epinephrine injection and endoluminal clipping. Comparison of unenhanced (**a**) and arterial-phase (**b**, **c**) CT images detected active contrast extravasation in the duodenal lumen (*arrowheads*) abutting the metallic clips (*thin arrows*), consistent with persistently ongoing arterial haemorrhage. Endovascular therapeutic embolisation of the gastroduodenal artery was necessary to control bleeding

**Fig. 10:**
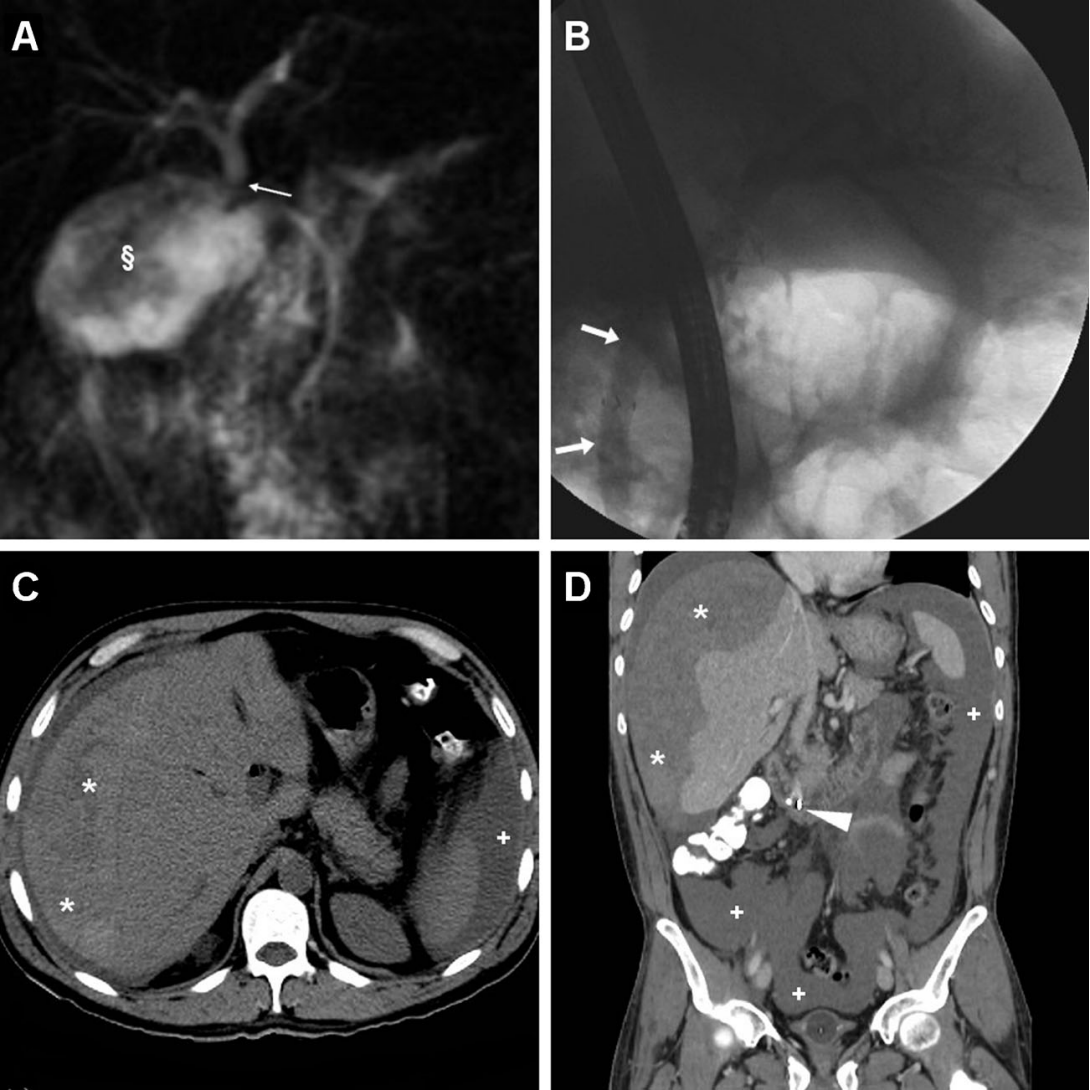
A 39-year-old male with previous Bismuth type II iatrogenic bile duct injury during laparoscopic cholecystectomy 10 months earlier, shown in a coronal thick-slab MR cholangiography (*MRCP*) image (*thin arrows in* **a**), which required immediate reintervention to remove the surgical clips plus temporary plastic biliary stenting. Note haematoma in the gallbladder fossa (§). Twenty-four hours after endoscopic positioning of the covered metallic biliary stent (**b**), he suffered from sudden abdominal pain, vomiting and hypotension, and unenhanced (**c**) CT images detected a large hyperattenuating subcapsular liver haematoma (*) and haemoperitoneum (+) attributed to guidewire manoeuvres, which progressed at repeated contrast-enhancement CT on the next day (**d**), thus dictating immediate surgical evacuation

Although clinically significant haemorrhage requiring angiographic, endoscopic or surgical intervention is rare (less than 1/1,000 sphincterotomies), CT imaging may help in the diagnosis by showing hyperattenuating fluid consistent with blood in the choledochus (Fig. 8) or duodenal lumen. Residual diluted CM in the duodenal or jejunal lumen may sometimes be confused with fresh haemorrhage. Alternatively, intramural bleeding may appear as high-attenuation duodenal wall thickening. Arterial-phase acquisition completed with maximum-intensity projection (MIP) reconstructions at vascular window settings may effectively show active contrast extravasation in the duodenum, indicating ongoing bleeding (Fig. 9). Furthermore, following operative ERCP bleeding may occasionally form intrahepatic or subcapsular hepatic haematomas with associated haemoperitoneum (Fig. 10) [13, 31, 32].

The CT detection of active bleeding represents an indication for endoscopic, interventional or surgical treatment. The generally accepted first-line treatment to control bleeding is repeated endoscopy with several haemostatic methods such as flushing with epinephrine solution, balloon tamponade, haemostatic (fibrin glue) injection, hemoclip placement, electrocoagulation or temporary stent placement. When endoscopic procedures fail, bleeding control requires interventional radiological embolisation or surgery [33–36].

## Duodenal perforation

Representing the rarest yet most feared occurrence, DP complicates 0.1–1 % of ERCP procedures and is associated with a non-negligible mortality rate (9–18 %). DP is mostly associated with standard (using an electrical papillotome catheter) or pre-cut (with a needle knife) sphincterotomy extending beyond the intramural CBD portion and with guidewire manipulation. Other risk factors for ERCP-related perforation include dilated CBD, stricture dilatation, SOD, presence of peripapillar diverticula and previous Billroth II surgery [2, 6–8, 14, 19, 37, 38].

**Fig. 11:**
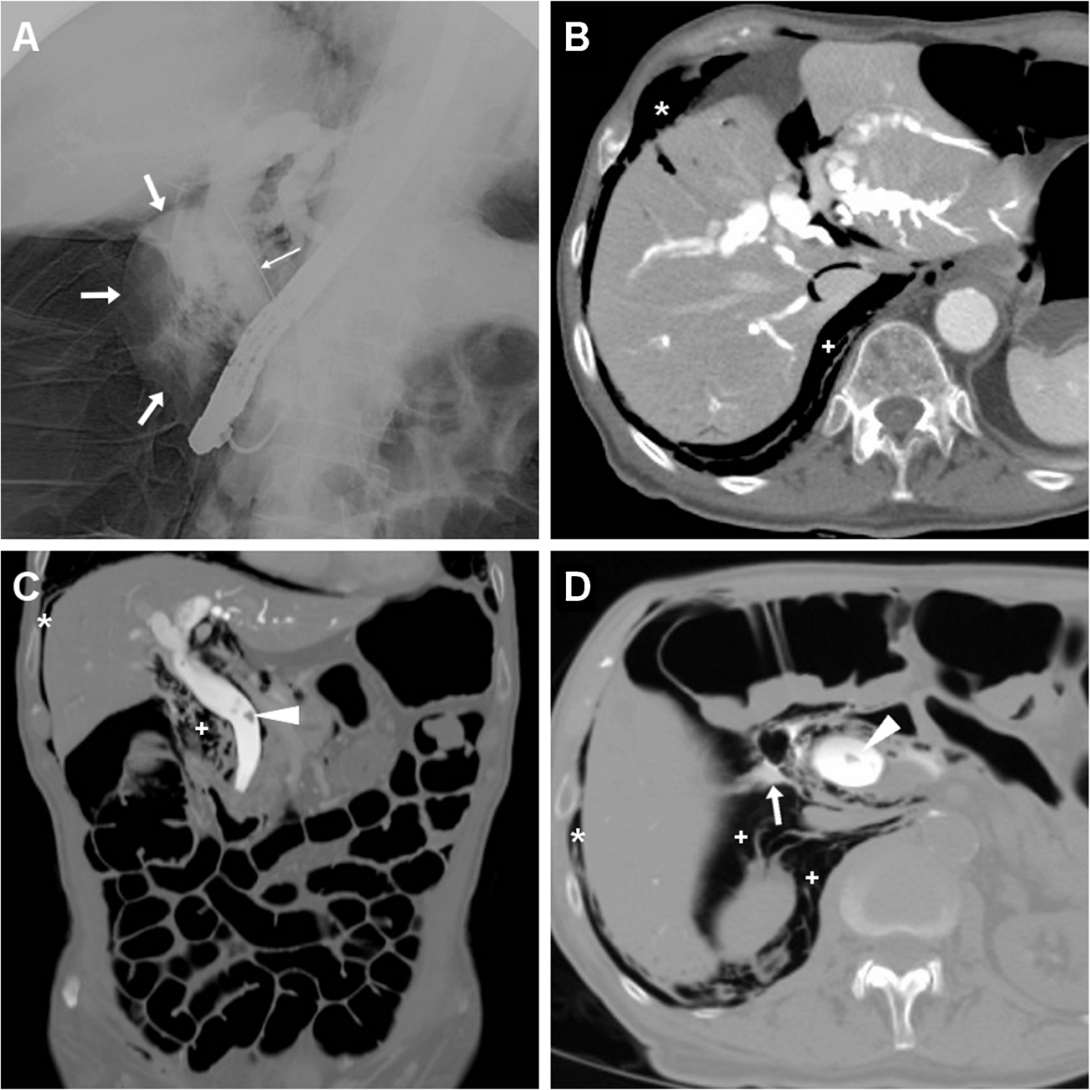
An elderly 80-year-old male with multiple comorbidities underwent endoscopic treatment of choledocholithiasis, including pre-cut sphincterotomy of the Vaterian papilla and stone retrieval using a basket device. During ERCP, fluoroscopy (**a**) showed opacification of dilated intrahepatic and common bile ducts and of a sizeable extraluminal CM collection (*thin arrows*) from lateral duodenal wall perforation (type I according to the Stapfer classification system). A few hours later CT (**b**–**d**) showed diffuse gaseous bowel distension from insufflation during endoscopy, presence of posterior pneumomediastinum, perihepatic and right parietocolic pneumoperitoneum (*), and extensive retroperitoneal emphysema in the right perirenal, posterior and anterior pararenal spaces (+). Note persistently opacified intrahepatic and common bile ducts with residual lithiasis fragments (*arrowheads*), persistently extravasated CM (*arrow* in **d**). Imaging findings and worsening clinical conditions with peritonitis dictated urgent laparotomy, which confirmed intra-abdominal free air, and included opening and toilette of common bile duct and positioning of a Kehr T-tube. The patient finally recovered after a prolonged hospital stay

In a variable proportion (26–48 %) of patients DP is suspected or verified during ERCP by means of CM injection through the endoscope showing extraluminal CM leakage (Fig. 11). Alternatively, clinical presentation occurs hours or days later, may be closely similar to those associated with PEAP and is not unusually mild compared to the imaging findings [2, 6–8, 14, 37, 38].

The classification system proposed by Stapfer et al. (Table 1) categorises DP into four classes in descending order of severity, on the basis of the mechanism and anatomical location, in order to predict the need for surgery [8].

Although controversy exists about the optimal approach, the management of ERCP-related DP should be individualised according to its type, clinical picture and imaging findings. Whereas type I (endoscope-related) duodenal wall perforations invariably require early surgery, the majority (approximately 70 %) of patients with peri-Vaterian (type II) DP tend to seal spontaneously and thus are amenable to endoclipping (Fig. 1) or conservative management (nasogastric drainage, nil-by-mouth and parenteral nutrition, intravenous hydration and antibiotics). Currently, the therapeutic approach to management of stable patients is increasingly nonsurgical and proves successful in approximately two-thirds of cases: unfortunately, delayed recognition of type I perforations and failure of conservative treatment are associated with a high death rate (50 %) from sepsis. As from the WSES guidelines, accepted indications for surgical treatment include clinical features such as sepsis and peritonitis, imaging features (free intraperitoneal air, periduodenal or retroperitoneal fluid collections, contrast extravasation at ERCP, CT or upper gastrointestinal study with water-soluble CM, retained stones or basket/wire endoscopic instruments) and failure of conservative management. Surgery may include perforation closure or duodenal exclusion, retroperitoneal drainage, CBD exploration and T-tube insertion [2, 6, 8, 12, 14, 38–42].

**Fig. 12:**
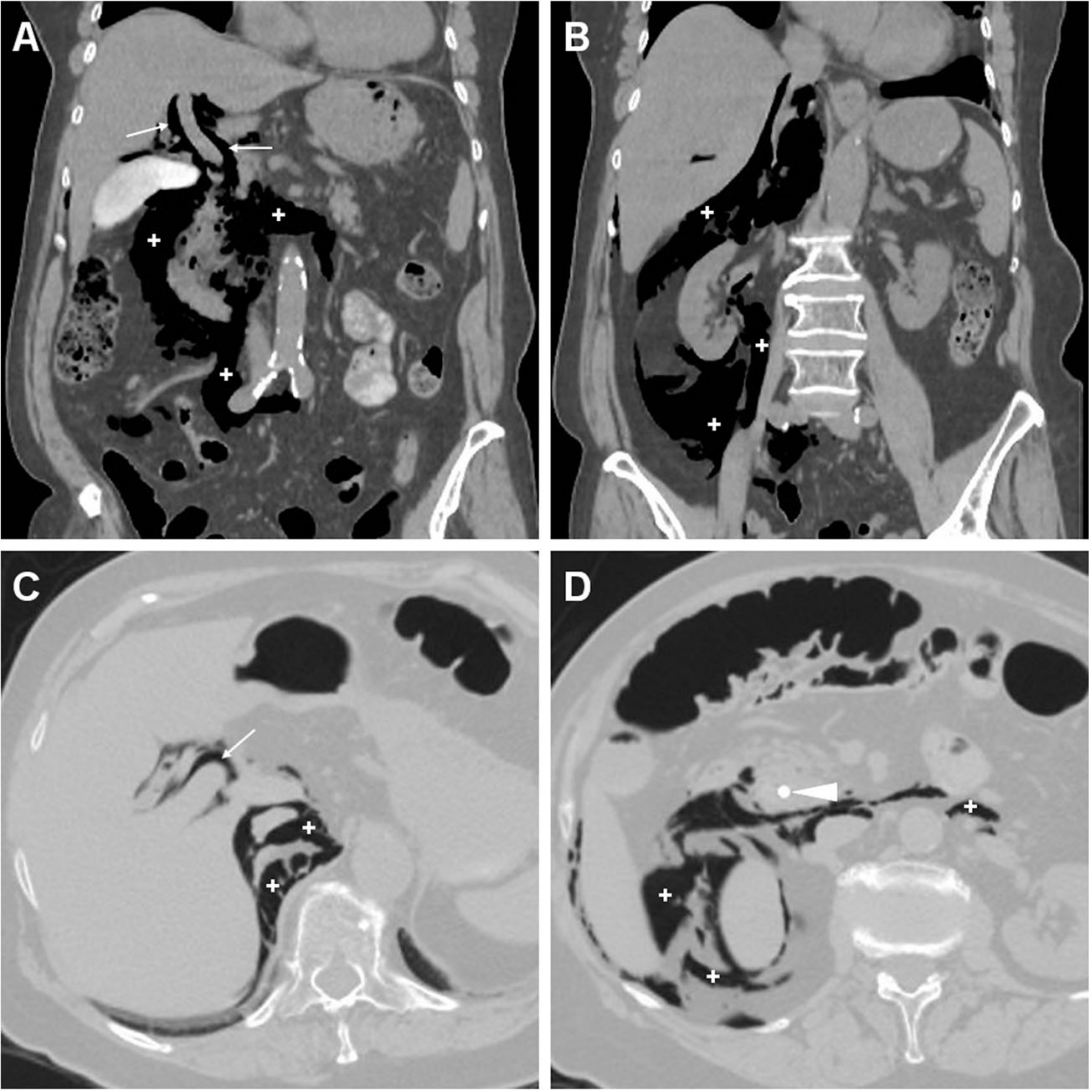
Severe retroperitoneal (type II) DP in an 80-year-old female following ERCP with sphincterotomy, incomplete removal of choledocholithiasis and positioning of a plastic CBD prosthesis (*arrowhead*). Post-procedural unenhanced CT viewed at both soft-tissue (**a**, **b**) and bone (**c**, **d**) window settings showed extensive retroperitoneal air (+) predominantly located in the right peri- and pararenal spaces, around the descending duodenum and tracking along the inferior vena cava and portal vein (*thin arrows*). Surgical exploration dictated by sepsis failed to detect a perforation site, and the patient recovered fully

**Fig. 13:**
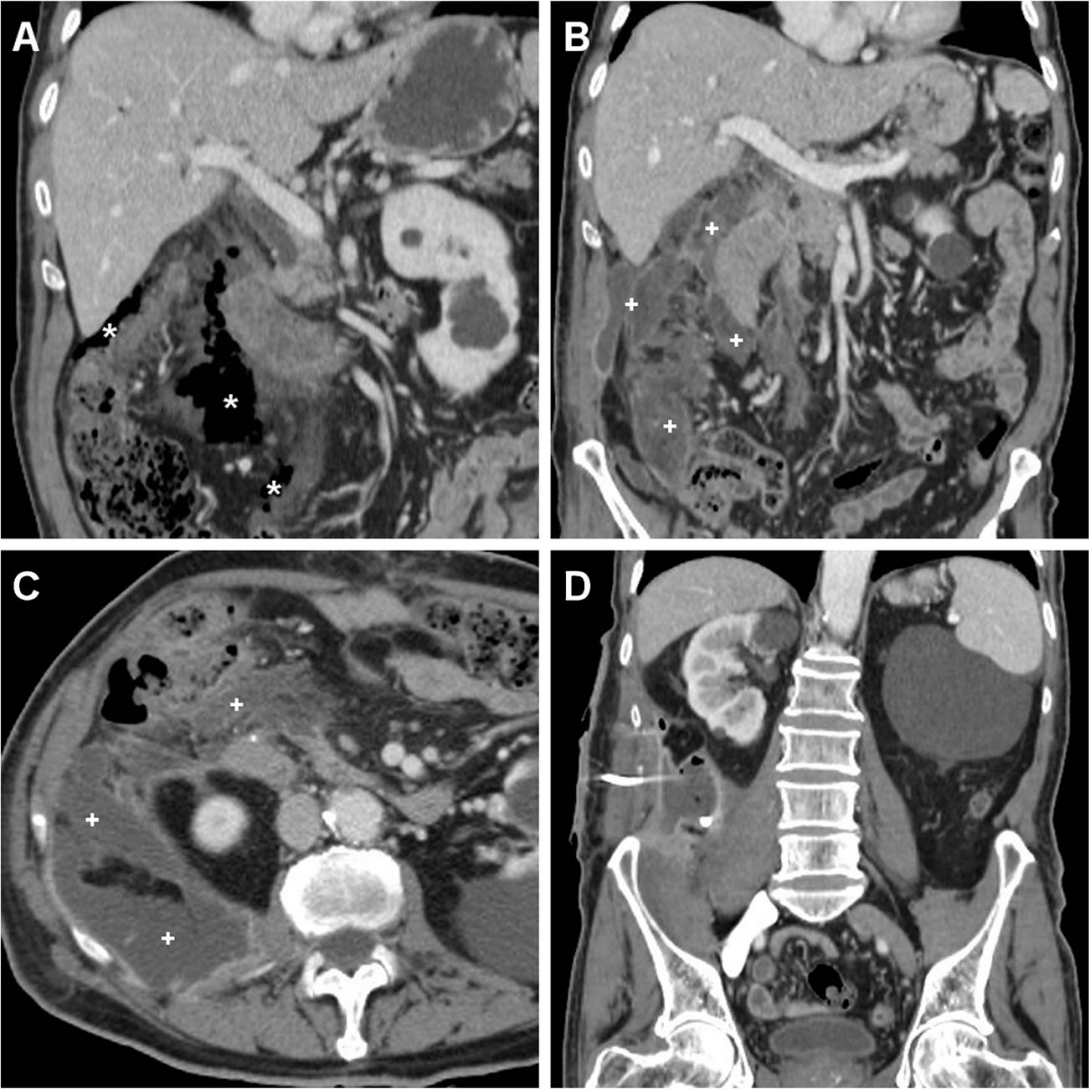
Failed conservative treatment of DP in an 83-year-old male with chronic duodenal stricture who underwent ERCP to prevent further episodes of infectious cholangitis. The next day, contrast-enhanced CT (**a**) showed moderate right-sided retroperitoneal gas (*). Follow-up CT (**b**, **c**) 4 days later showed formation of multiple confluent periduodenal and right parietocolic fluid-like infected collections (+), with extensive occupation of the ipsilateral posterior pararenal space, which required percutaneous drainage (**d**) to relieve sepsis

**Fig. 14:**
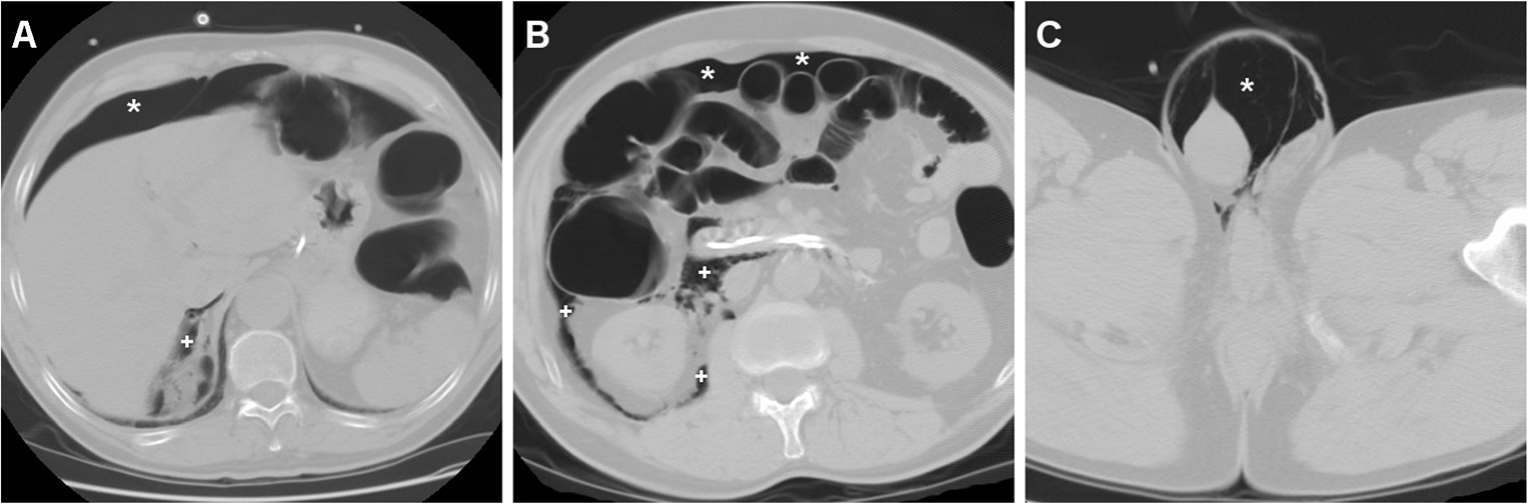
Stapfer type I DP with moderate retroperitoneal air (+) plus pneumoperitoneum extending to the scrotum (*) shown by emergency unenhanced CT in a 62-year-old male undergoing ERCP treatment of choledocholithiasis. Laparotomic surgery included duodenotomy and suture of a posterior duodenal wall discontinuity, toilette and drainage of the peritoneal cavity. The subsequent course was uneventful and repeated CT with oral CM at discharge (*not shown*) excluded extraluminal contrast leak

The CT imaging hallmark of DP is represented by extraluminal air, which may sometimes be located in the duodenal wall, but most commonly dissects through the retroperitoneal compartments. Due to the anatomic location of the CBD, the typical appearance of a type II DP includes air collecting posteriorly to the duodenum and pancreatic head, in the anterior pararenal and right perirenal space, surrounding the inferior vena cava, portal vein and splanchnic vessels, and sometimes crossing the midline (Figs. 11, 12, 13 and 14). Occasionally, air may track cranially in the posterior mediastinum [2, 8, 9, 13, 38].

However, the presence and amount of retroperitoneal air at imaging do not linearly correlate with the severity of the injury or with the need for invasive treatment since it rather reflects the degree of continuous endoscopic air insufflation and manipulation after an undetected injury occurred. Therefore, successful nonoperative management of type II DP with extensive retroperitoneal air is possible, provided that the patient does not develop peritonitis, fever or impending shock. However, negative physical findings do not reliably exclude surgery since severe DP may be masked clinically by the retroperitoneal site especially in elderly or chronically ill patients. Extraluminal CM leak (Fig. 11), pneumoperitoneum (Fig. 14) and fluid collections represent the key imaging findings, which usually shift the therapeutic approach towards surgery. During conservative treatment, repeat CT may be warranted after a 48–72-h interval to confirm sealed leaks and absent fluid collections as well as to monitor the patient until recovery [2, 6, 12, 13, 15].

## Post-procedural infections

Although the current guidelines recommend antibiotic prophylaxis in known or suspected biliary obstruction, bacteraemia is a common event (15–27 %) after diagnostic or therapeutic ERCP. The reported incidence of clinically significant iatrogenic infections is limited (1–3 %), but sepsis represents a common cause of death. Specific risk factors for infection include stenting across tumours, obstructed ducts and jaundice, combined percutaneous-endoscopic procedures, primary sclerosing cholangitis, incomplete or failed biliary drainage [2, 6].

**Fig. 15:**
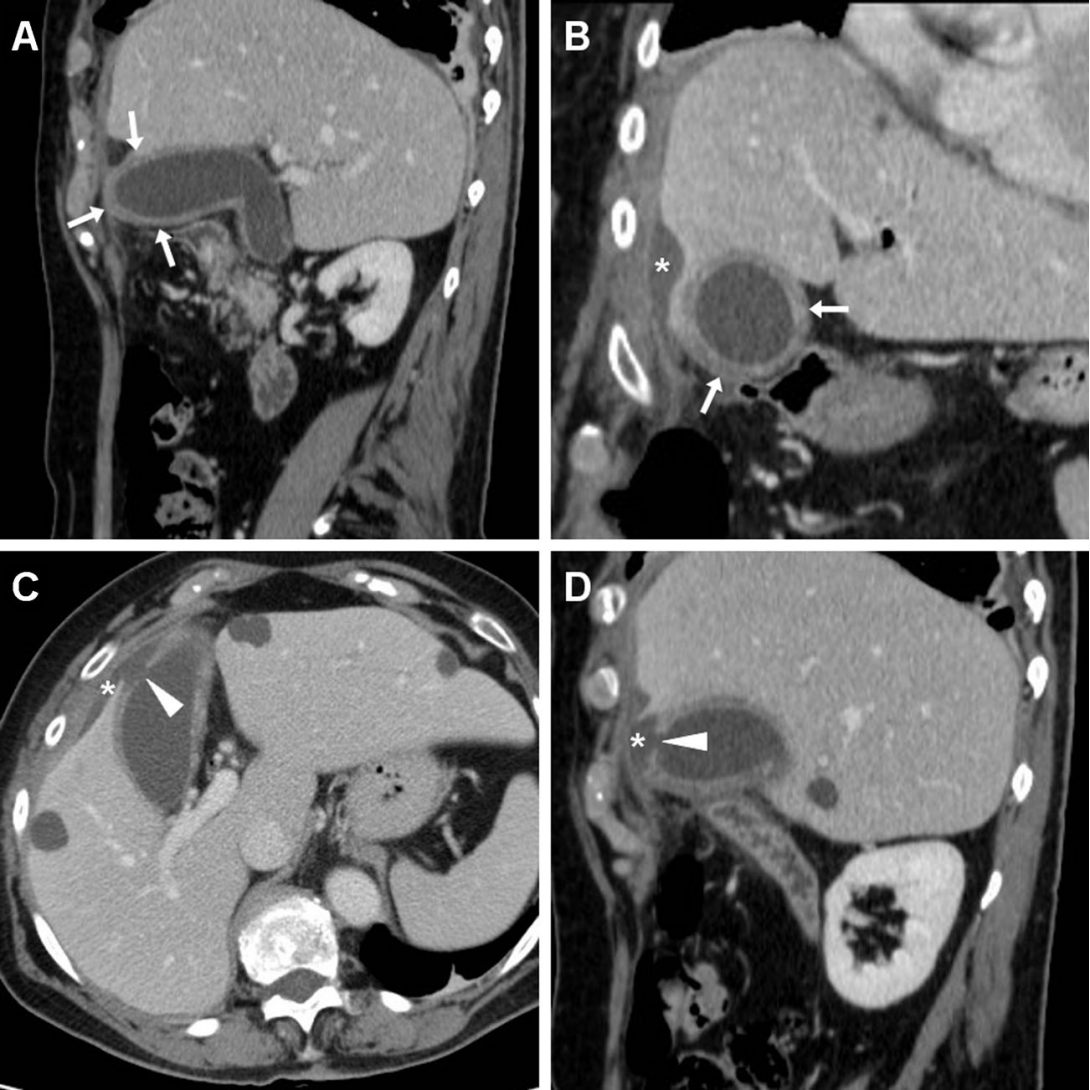
Acute cholecystitis in a 66-year-old female manifesting with fever and transverse upper abdominal pain 3 days after ERCP with sphincterotomy and biopsy of ampullary adenoma including antibiotic prophylaxis. Multiplanar contrast-enhanced CT images show a thickened gallbladder wall (*arrows*) with focal perforation (*arrowhead* in **c**) and localised collection (*), which required emergency laparotomic cholecystectomy.

**Fig. 16:**
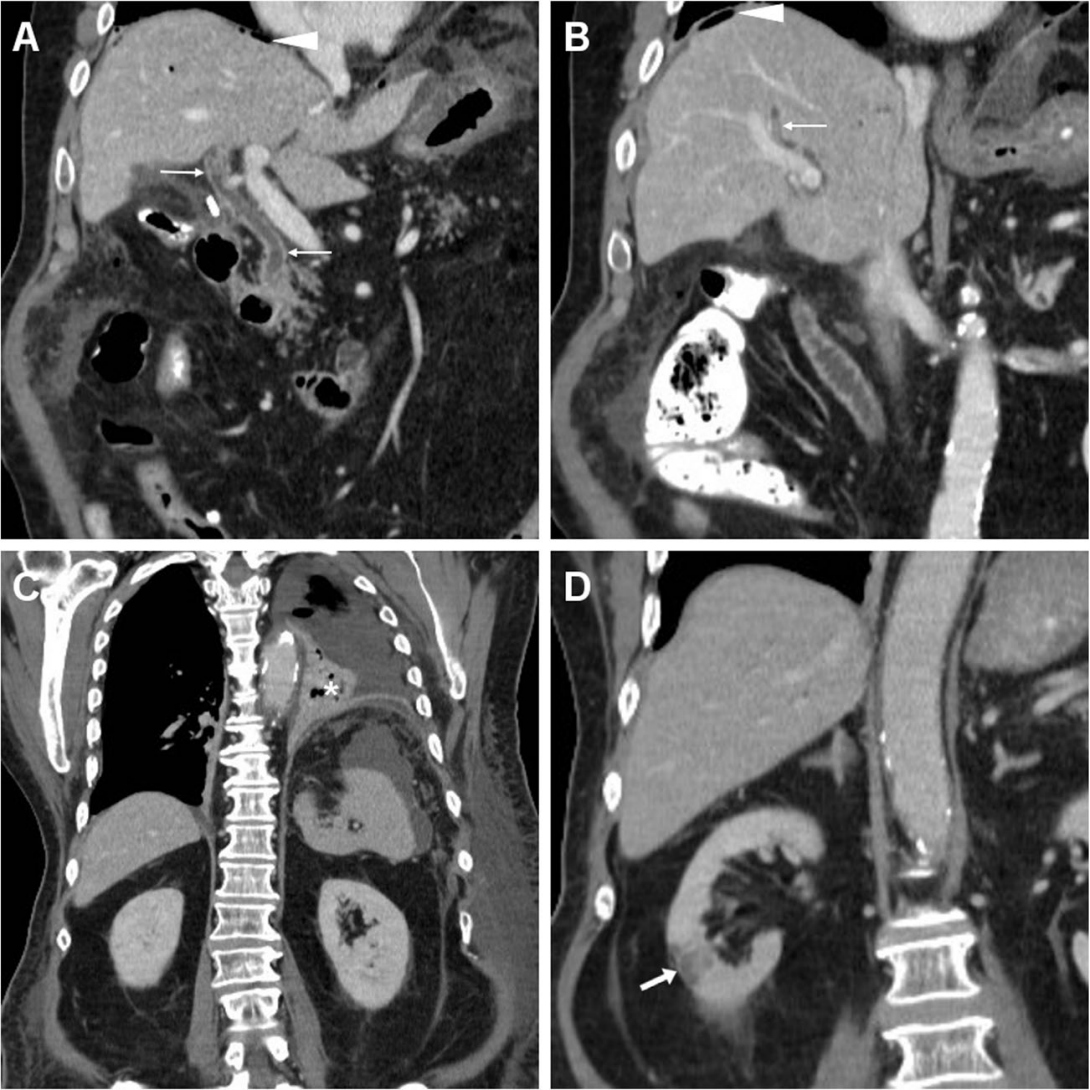
Cholangitis and sepsis in an 81-year-old female following endoscopic stone removal using a Fogarty catheter. Multiplanar contrast-enhanced CT images showed minimal pneumoperitoneum (*arrowheads*) and peritoneal effusion, thickened enhancing walls of CBD and main intrahepatic ducts (*thin arrows*). Additionally, left-sided pneumonia (* in **c**) and a limited renal infarct (*arrow* in **d**) were seen. Worsening clinical conditions dictated surgery, which revealed biliary peritonitis from contained cystic duct injury. The patient recovered after a long intensive care unit stay

**Fig. 17:**
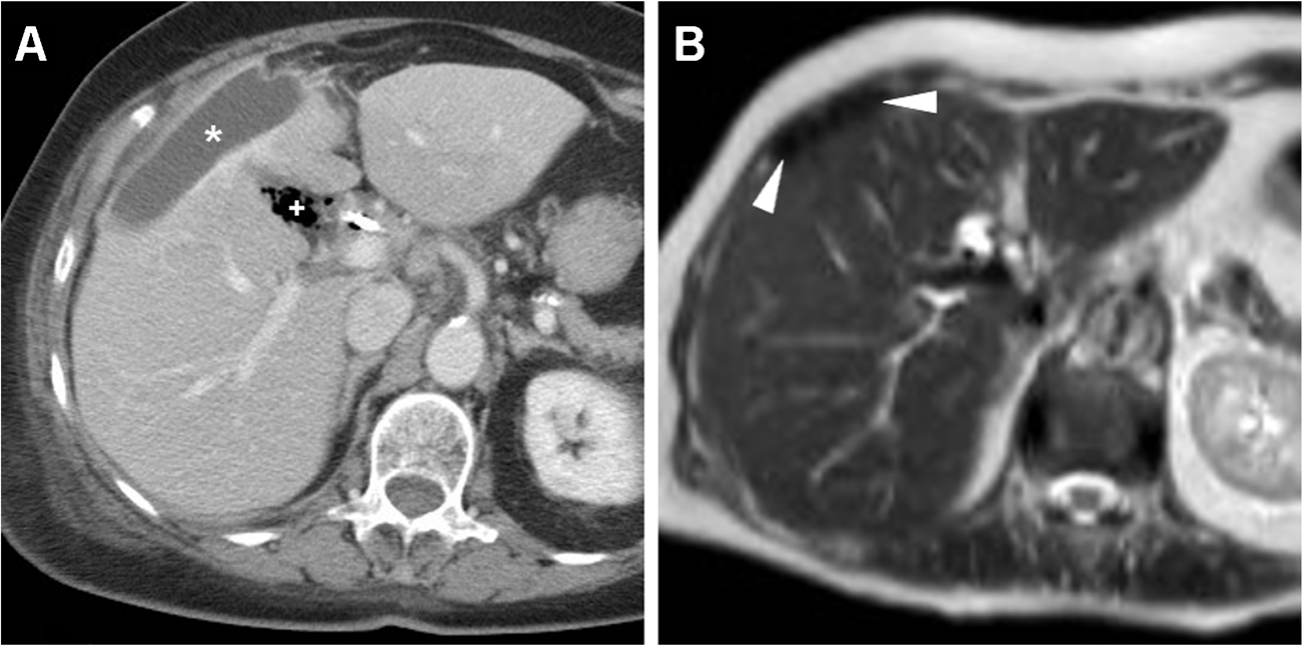
Perihepatic abscess diagnosed 10 days after endoscopic stent positioning through a cytologically benign distal CBD stricture in a 78-year-old female. Contrast-enhanced CT (**a**) requested because of fever and right hypochondrium pain revealed a fluid-like collection (*) with an enhancing peripheral rim and minimal extraluminal air (+) predominantly in the right-sided retroperitoneum. Urgent surgery included cholecystectomy and abscess drainage. Postoperatively, unenhanced T2-weighted MRI (**b**) depicted resolution of the abscess (*arrowheads*)

**Fig. 18:**
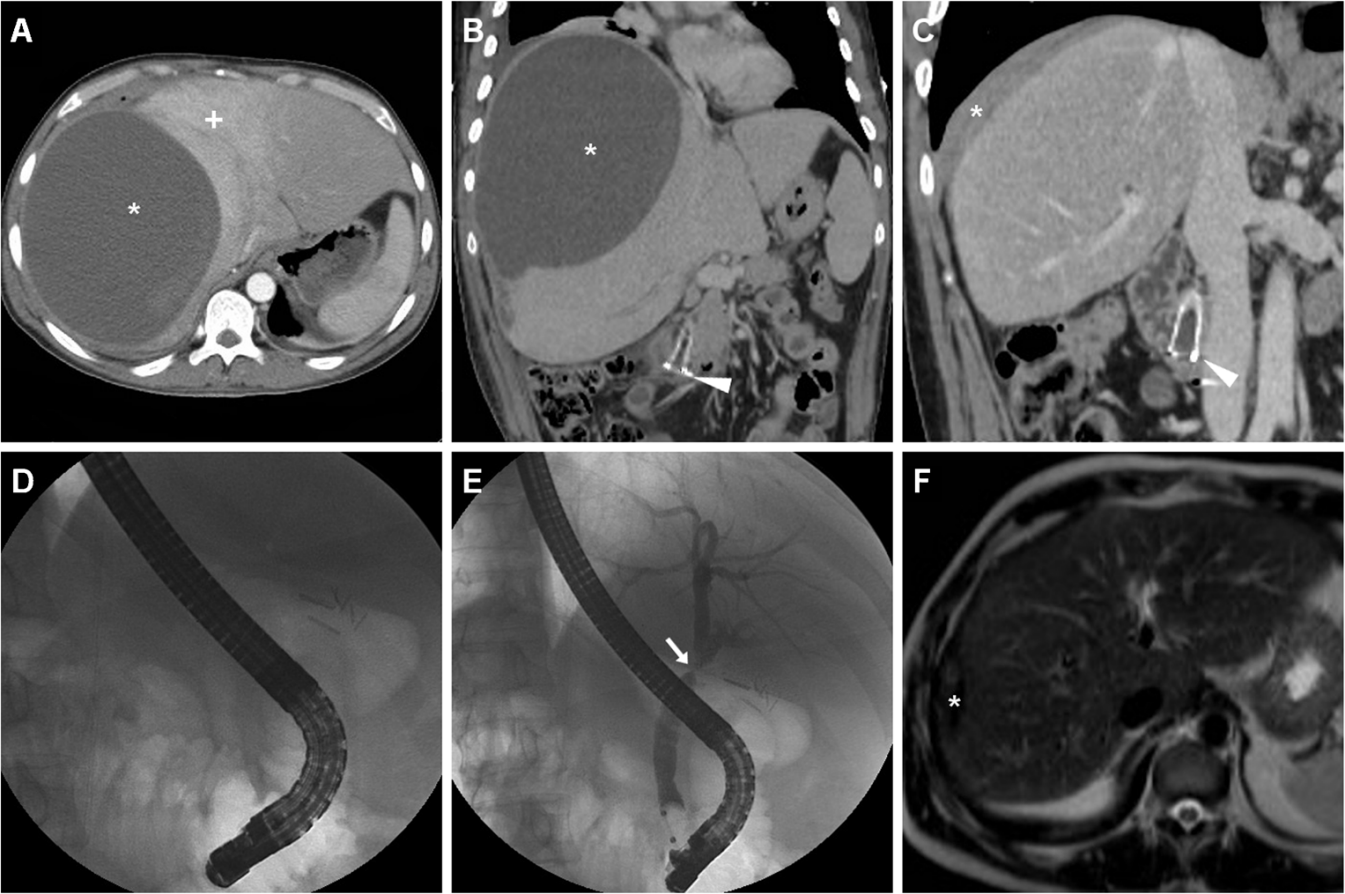
One month after evacuation of the haemoperitoneum and subcapsular hepatic haematoma, the same patient in Fig. 10 suffered form right-sided thoraco-abdominal pain and fever. Arterial- (**a**) and venous-phase (**b**) CT showed a huge, fluid-attenuating (15 HU) subphrenic collection (*) with inflammatory enhancement of the surrounding, compressed liver parenchyma (+) and thin peripheral enhancement, consistent with an abscess. Ipsilateral pleural effusion and basal lung atelectasis were associated. Percutaneous drainage yielded 3 l of stinking pus. Note the correctly positioned metallic CBD stent (*arrowheads*), and clinical, laboratory and imaging (* in **c**) resolution was obtained. During endoscopic replacement, the biliary stent was not found anymore (**d**), displaced and probably lost with stools. Endoscopic cholangiogram (**e**) confirmed persistent iatrogenic biliary stricture (*arrow*). Follow-up MRI (T2-weighted image in **f**) showed resolved subphrenic abscess with low signal intensity consistent with fibrosis (*)

The spectrum of post-ERCP infectious complications encompasses cholecystitis, septic cholangitis, liver abscess and systemic sepsis. Occurring in approximately 0.2–0.5 % of patients, cholecystitis has been correlated to cholelithiasis and intraprocedural filling of the gallbladder with CM, and it is heralded by the appearance of luminal overdistension and circumferential mural thickening (Fig. 15). Septic cholangitis is suggested by thickened enhancing CBD and intrahepatic duct walls (Fig. 16). Accessory signs of sepsis such as atelectasis/pneumonia, splenic or renal infarcts may be observed (Fig. 16). Alternatively, sepsis may result from superinfection of PNPFCs or abnormal collections from DP (Fig. 13). Abscess collections usually display enhancing mural thickening, similar attenuation to that of non-infected fluid collections and sometimes internal gas bubbles (Figs. 17 and 18) [6, 13, 15].

## Stent-related complications

**Fig. 19:**
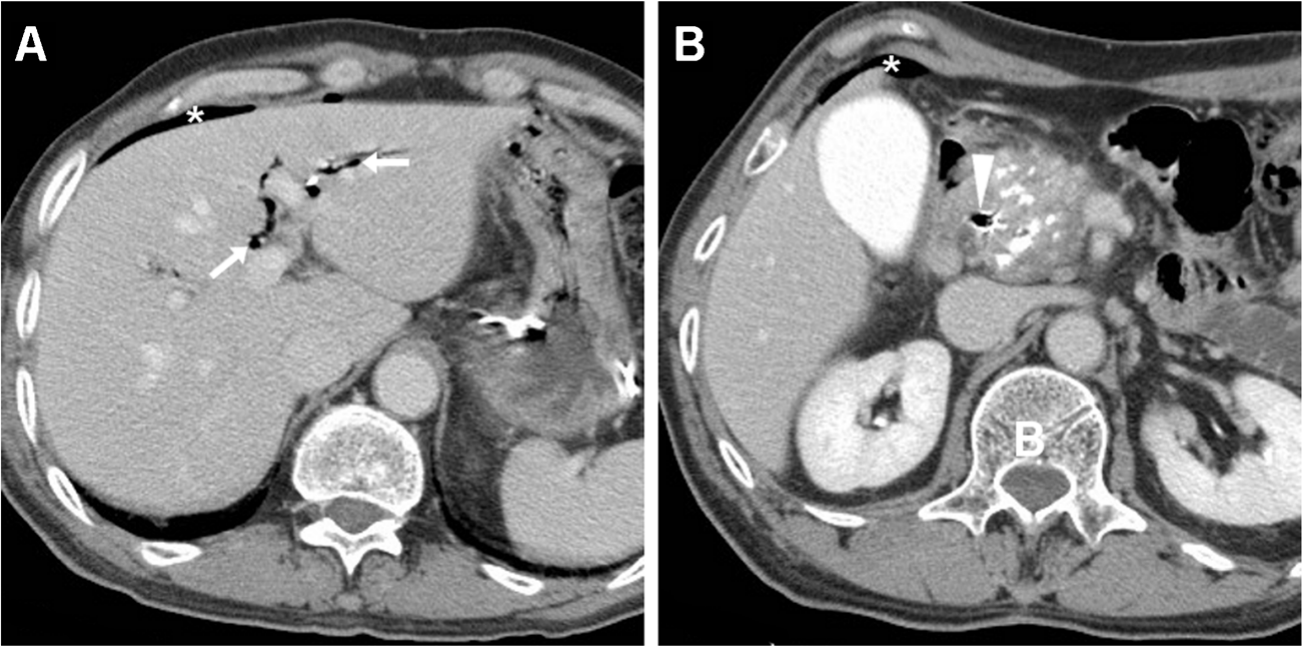
In a 60-year-old male with severe chronic calcific pancreatitis, after ERCP positioning of the metallic pancreatic stent (*arrowhead* in **a**) contrast-enhanced CT showed minimal intraperitoneal air (*), which was treated conservatively with success. Note pneumobilia (*arrows*) and retained CM in the gallbladder

Relatively rare, early complications after biliary stenting include haemorrhage (Fig. 10), PEAP, stent misplacement, perforation (Fig. 19) and injury to the CBD or main pancreatic duct [13, 17].

Chronic stent-related complications are more common and include stent obstruction (25–35 % of patients), migration, fracture or collapse, infections such cholangitis, liver abscess (Fig. 18) or sepsis. Biliary stent dislodgement (Fig. 18) occurs after a variable time interval in up to 6 % of patients and is more frequent with plastic stents compared to SEMS. Displacement may be proximal, particularly in patients with malignant obstruction, or alternatively distal to the intestine, the latter not unusually asymptomatic [4, 5, 17, 43, 44].

## Conclusion

Diagnosis and management of post-ERCP complications should combine clinical and laboratory data with imaging appearances. Urgent CT imaging is crucial to promptly identify and categorise the most common and unusual complications after ERCP, particularly to triage those occurrences that require prolonged in-hospital treatment or surgical reoperation. Knowledge of ERCP procedural details, the appropriate CT acquisition technique and familiarity with normal appearances are needed to diagnose and grade oedematous or necrotic PEAP, bleeding, DP and infections, which may have similar, nonspecific clinical and laboratory manifestations [2, 6, 12].
